# II Consensus of the Brazilian Society of Dermatology for the treatment of alopecia areata^☆^

**DOI:** 10.1016/j.abd.2024.10.001

**Published:** 2024-12-04

**Authors:** Paulo Müller Ramos, Alessandra Anzai, Bruna Duque-Estrada, Daniel Fernandes Melo, Flavia Sternberg, Leopoldo Duailibe Nogueira Santos, Lorena Dourado Alves, Fabiane Mulinari-Brenner

**Affiliations:** a Department of Infectology, Dermatology, Imaging Diagnosis and Radiotherapy, Faculty of Medicine, Universidade Estadual Paulista, Botucatu, SP, Brazil; b Department of Dermatology, Faculty of Medicine, Universidade de São Paulo, São Paulo, SP, Brazil; c Hair Studies Center, Instituto de Dermatologia Prof. Rubem David Azulay, Santa Casa da Misericórdia do Rio de Janeiro, Rio de Janeiro, RJ, Brazil; d Department of Dermatology, Universidade do Estado do Rio de Janeiro, Rio de Janeiro, RJ, Brazil; e Department of Dermatology, Faculty of Medicine, Universidade Federal de São Paulo, São Paulo, SP, Brazil; f Department of Medicine, Santa Casa de São Paulo, São Paulo, SP, Brazil; g Department of Dermatology and Allergology, Hospital do Servidor Público Municipal, São Paulo, SP, Brazil; h Department of Medicine, Universidade de Taubaté, Taubaté, SP, Brazil; i Department of Tropical Medicine and Dermatology, Universidade Federal de Goiás, Goiânia, GO, Brazil; j Department of Internal Medicine, Universidade Federal do Paraná, Curitiba, PR, Brazil

**Keywords:** Adrenal cortex hormones, Alopecia areata, Consensus, Janus Kinase inhibitors, Methotrexate, Therapeutics, Triamcinolone

## Abstract

**Background:**

Alopecia areata is a highly frequent disease with great variability in clinical presentation, severity, and prognosis. It has a significant negative impact on quality of life, especially in the moderate and severe forms.

**Objective:**

To disseminate guidelines, prepared by a group of Brazilian experts, for the treatment and follow-up of patients with alopecia areata.

**Methods:**

Eight specialists from different university centers with experience in alopecia areata were appointed by the Brazilian Society of Dermatology to reach a consensus on its treatment. Using the adapted DELPHI methodology, relevant elements were considered and then an analysis of the recent literature was carried out and the text produced. Consensus on the guidelines was defined with the approval of at least 70% of the panel of experts.

**Results/Conclusions:**

Treatments vary according to patient age and disease severity. Intralesional injectable corticosteroid therapy was considered the first option for localized disease in adults. In severe cases, Janus Kinase inhibitors are the treatment with the highest level of evidence. Systemic corticosteroid therapy and immunosuppressants (corticosteroid-sparing agents) are also options in these cases. Contact immunotherapy (diphencyprone) is an alternative for stable extensive cases. The assessment of side effects is as important as the hair regrowth rate.

## Introduction

Alopecia areata (AA) is an autoimmune disease that targets hair follicles in the anagen phase and is a common cause of non-cicatricial alopecia. AA usually manifests before the age of 40, with no gender or ethnic predilection. In a survey conducted by the Brazilian Society of Dermatology (SBD, *Sociedade Brasileira de Dermatologia*), AA was responsible for 1.2% of all dermatological consultations. Among the causes of hair loss, it was only less frequent than androgenetic alopecia and telogen effluvium.^1^ The risk of developing AA during ones lifetime is estimated at 2%, but severe forms are less common. The overall prevalence of total alopecia areata (TA) in the population is 0.08% and that of universal alopecia areata (UA) is 0.02%.^2^, ^3^

Since 2020, when the SBD Consensus on the treatment of AA was published,^4^ several important studies have been published on the subject, notably in relation to janus kinase inhibitors (JAKi) in the treatment of severe AA.^5^, ^6^ Currently, two of these drugs, baricitinib and ritlecitinib, are approved by ANVISA for use in Brazil.

These developments have had a major impact on the treatment of AA, which made it essential to update the text published in 2020. The objective of this study is to update the treatment and follow-up guidelines for patients with AA based on the recommendations of a group of Brazilian experts.

## Methods

Eight dermatologists from different university hospitals, with experience in AA, were appointed by the SBD to develop guidelines for the treatment of AA. The adapted DELPHI method was used for this purpose. The previously published consensus^4^ was reviewed and new recommendations were added, based on the literature update.

The first step consisted of reviewing the structure of the text and the topics to be addressed. Then, the topics were divided among the participants, who carried out the literature review and drafted the text. Subsequently, all sections and recommendations on the treatment of AA were reviewed and discussed among all the panel members. The consensus for the recommendations was defined with the approval of at least 70% of the panel.

## Assessment of extent and severity

Although AA has historically been classified into different clinical patterns (Table 1), treatment is based on severity.^7^, ^8^, ^9^ To assess severity, the first step is to determine the extent of disease involvement on the scalp. The Severity of Alopecia Areata Tool (SALT) is a score that represents the percentage of the scalp area affected. A SALT score of 0 indicates a patient with no AA-induced hair loss, while a SALT score of 100 indicates a patient with alopecia of the entire scalp.^10^

**Table 1:** Clinical forms of AA.

**Patch AA**	Circumscribed areas of alopecia, oval or rounded; single or multiple. Although the elementary lesion is not actually a patch, the term became established by the free translation of patch from English.
**Total AA**	Alopecia affecting the entire scalp.
**Universal AA**	Alopecia affecting the entire body.
**Ophiasis AA**	Band-like alopecia on the lateral and occipital regions of the scalp.
**AA Sisaipho (Ophiasis inversus)**	Alopecia in the fronto-parietal area, sparing the lateral and occipital regions.
**Diffuse AA**	Global decrease in density, without patches
**Diffuse acute and total AA**	Diffuse, progressive and rapid hair loss, usually progressing to total AA within three months.

**Fig. 1:**
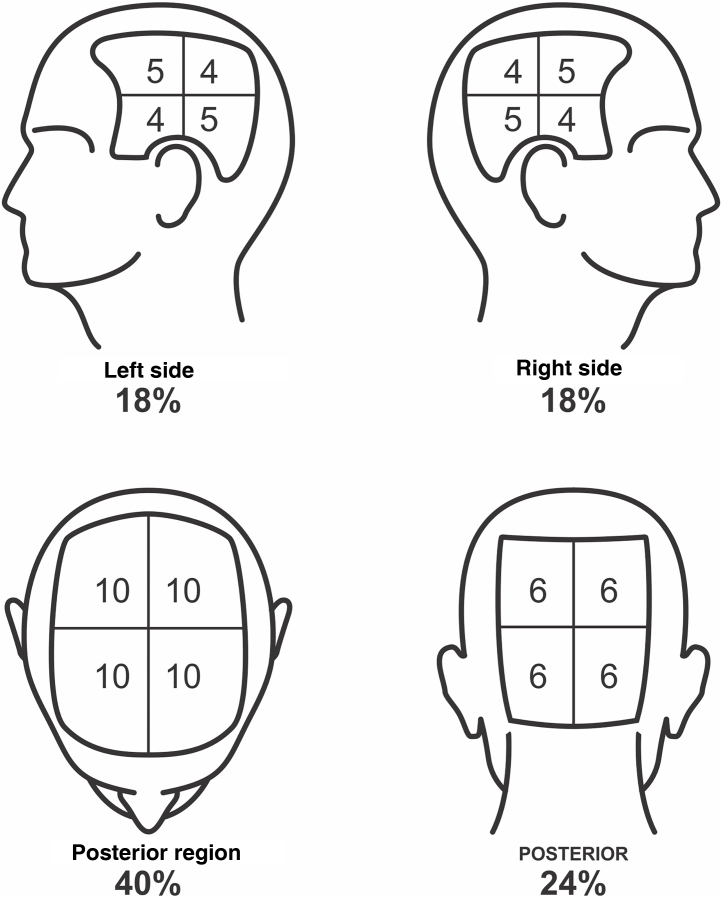
SALT score for determining the area of ​​hair loss secondary to AA. The image shows the percentage of each region and the subdivision of these into quadrants. The evaluator must estimate the area of ​​alopecia in each region and add them together. Areas of alopecia include those with no hair or thin hair, and areas that are necessarily re-grown with terminal hair. The SALT score will result from the sum of these values. For example, a patient with alopecia that affects half of the upper region and a quarter of the posterior region will have a SALT score of 20 + 6 = 26.

**Fig. 2:**
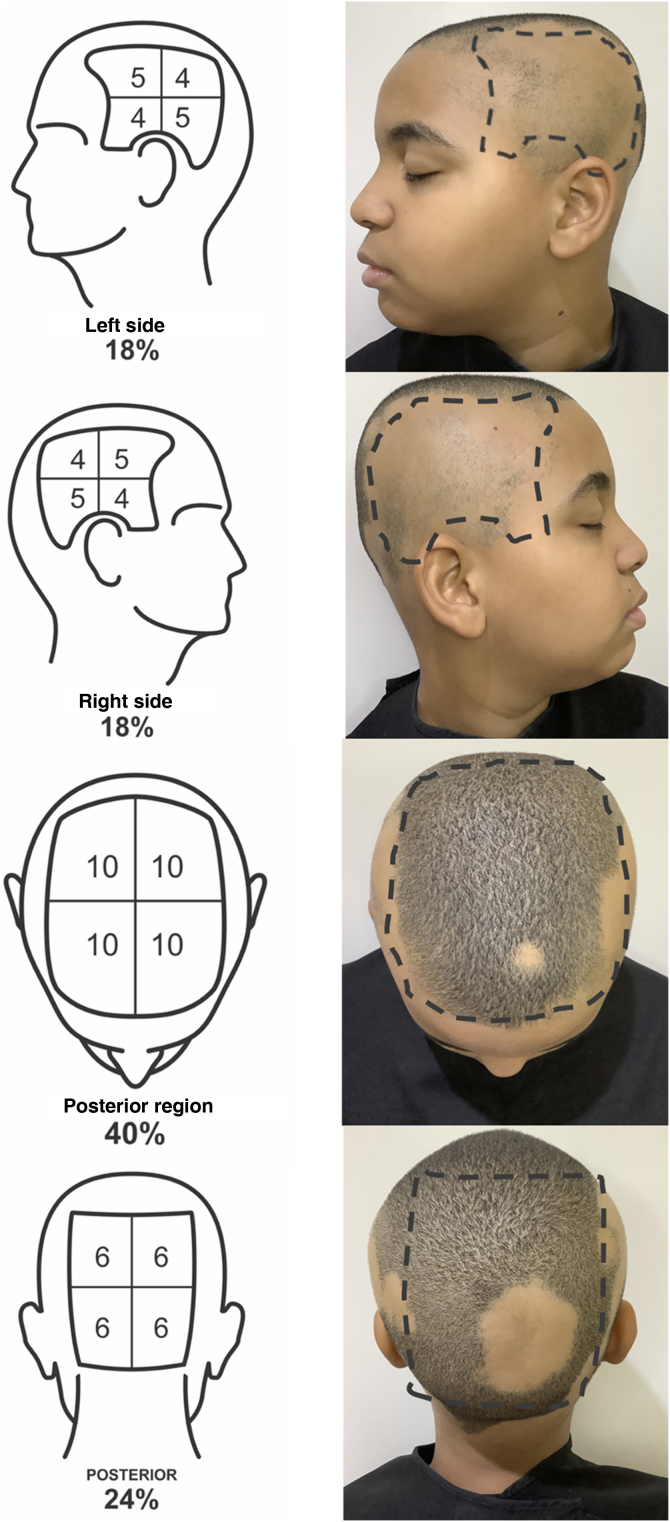
Example of the SALT Score evaluation in a patient with alopecia areata: 14 (left side) +14 (right side) +2 upper region +6 posterior region = SALT Score 36.

To define the SALT score, the scalp should be divided into four regions: posterior, upper, and two lateral regions. The upper region represents an area of ​​40% of the scalp, the lateral regions, 18% each, and the posterior region, 24%. The evaluator should estimate the area of ​​alopecia in each of these regions and add them together. The result is the patient SALT score (Fig. 1, Fig. 2).

As proposed by King et al. in 2022 to classify severity, one considers mild cases as those affecting up to 20% of the scalp; moderate, from 21% to 49%, and severe, above 50%.^11^ Mild or moderate cases increase in severity by one level if one or more of the following additional criteria are present: noticeable involvement of eyebrows or eyelashes, inadequate response after at least six months of treatment, diffuse (multifocal) positive pull test, and a negative impact of AA on psychosocial functions^11^ (Table 2).

**Table 2:** AA severity scale.

**Severity**	**Extent of the alopecia area**
**Mild Areata**	20% or less of the scalp
**Moderate Areata**	21% to 49% of the scalp
**Severe Areata**	50% to 100% of the scalp
In cases of mild or moderate AA, increase the AA severity rating by one level if one or more of the following are present:	
Negative psychosocial impact resulting from AA	
Noticeable involvement of eyebrows or eyelashes	
Inadequate response after at least six months of treatment	
Multifocal positive pull test	

Adapted from King BA et al. 2002.^11^

Calculating the extent of AA and classifying severity are important steps in decision-making regarding the several therapeutic possibilities, which will be discussed below.

## Prognosis

AA is a chronic disease with an unpredictable course. Mild cases may show spontaneous remission, as well as develop into forms that are unresponsive to treatment.^7^ Approximately 50% of patients with single-patch AA have spontaneous hair regrowth in the first six months and 70% have regrowth in the first year but with the possibility of relapse after remission.^12^

Extensive forms of the disease and the ophiasis form generally do not respond well to treatment.^11^ Approximately 7% of the patients progress to the TA or UA subtypes.^7^ For severe cases, spontaneous recovery rates are less than 7%.^5^, ^6^ Other factors are indicative of worse prognosis of AA: onset in childhood, duration greater than 12 months, ungual involvement, association with atopy, association with autoimmune diseases, and family history of AA.^12^, ^13^, ^14^, ^15^

The time between episodes is unpredictable and they may even not recur. In localized/mild forms of the disease, permanent maintenance treatment is not necessary to prevent recurrence, unlike in severe forms, where interruption usually leads to disease relapse.^13^

## Complementary exams

The diagnosis of AA is made based on clinical and dermoscopic evaluation; histopathology may be performed in dubious cases. Complementary exams are not mandatory for the diagnosis. Depending on the clinical suspicion, the following may be requested: complete blood count, fasting blood glucose, antinuclear antibody (ANA), serological tests for syphilis, thyroid stimulating hormone (TSH), free T4, anti-thyroid peroxidase, anti-thyroglobulin, 25-OH vitamin D, vitamin B12, zinc, ferritin and C-reactive protein.

## Psychosomatic aspects

A good consultation should offer psychological support and discussion of the emotional aspects of the patient, and caregivers in pediatric cases. The psychological and social impact of hair goes beyond its biological meaning.^16^ Negative effects of AA on social, emotional well-being and mental health have been evidenced by quality of life indices. Self-image, interpersonal relationships, work or school activities can be affected by AA, even in patients with localized disease. More than half of the patients believe that the disease has major consequences in their lives.^17^ AA should not be dismissed as “just hair loss” or a “cosmetic” condition. Psychiatric diagnoses such as depression, anxiety disorder, adjustment disorder, or paranoia have been reported in up to 78% of patients.^18^, ^19^ AA is the second most common skin condition referred to psychiatrists by dermatologists, surpassed only by psoriasis.^20^

The efficacy of antidepressants, psychotherapy, relaxation techniques, and individual or group therapy in the treatment of AA has not been evaluated in clinical trials.^16^ More than half of the patients with AA believe that their behavior could determine whether their disease improves or worsens.^17^ To understand and respond to the difficulties that chronic AA can present, patients construct their own models of their condition. Information received from different sources, including doctors, family, friends, the internet, and existing social and cultural concepts about health and disease, shape this model. These beliefs motivate treatment. If the belief system is inadequate, poor adherence and abandonment of treatment are common.^16^

Support groups can be very important by providing identification and validation of emotions. Patients and caregivers share positive coping strategies for daily difficulties, such as getting hair prostheses, how they deal with situations of discomfort, as well as updates on research with a view to the future of the disease.

## Treatment

One of the essential steps in the treatment of AA is to explain to the patient the nature and course of the disease, as well as the available therapies. A realistic discussion of expectations is necessary.^7^ Due to the variable efficacy of treatments and their respective side effects, dermatologists have an important role in increasing patient’s awareness of the positive and negative aspects of each option.^7^ None of the therapies have been proven to modify the course of the disease in the long term, but in phase III clinical trials conducted with JAKi, it was observed that episodes of disease lasting less than four years were associated with a better clinical response.^21^

For mild cases, intralesional corticosteroid therapy is the treatment of choice. Topical corticosteroids, in combination with topical minoxidil, anthralin, and immunotherapy are alternatives. For moderate AA, in addition to the treatments described above, the combined use of oral minoxidil, systemic corticosteroid therapy for short periods, or other immunosuppressants may be considered. For severe AA, JAKi (baricitinib and ritlecitinib) are the drugs of choice (Table 3), and oral minoxidil can be used as an adjuvant. Each of these treatments will be detailed below, as well as the particularities of AA treatment in childhood and in special regions.

**Table 3:** Treatment of AA according to severity.

**Mild**	**Moderate**	**Severe**
**Intralesional corticosteroids**	In addition to the options for mild AA, consider:	**JAKi** in combination with oral minoxidil or topical/intralesional corticosteroids
**Topical corticosteroids**	**Short-term systemic corticosteroid therapy**	
**Topical anthralin**		**Systemic corticosteroids** in cases of rapid progression
**Topical immunotherapy**	**Systemic immunosuppressants**	
**Associate topical Minoxidil**		**Immunosuppressants** in the unavailability of JAKi
	**Associate oral Minoxidil**	

AA, Alopecia Areata; JAKi, Janus Kinase Inhibitors.

### Intralesional corticosteroid therapy

Intralesional corticosteroid therapy is recommended as the first option for adults with mild to moderate AA. Additionally, it can be used as an adjuvant in patients with severe disease. This route of administration crosses the epidermal barrier, making the drug available directly to the inflamed area.^22^, ^23^, ^24^ This minimizes the possible adverse effects of systemic corticosteroid therapy and provides greater drug penetration when compared to the topical route.^24^ Patients with isolated, small-sized alopecia patches (<3 cm) occupying less than 25% of the scalp are the best candidates for intralesional injection.^8^, ^22^, ^25^, ^26^

Approximately 60% to 75% of patients with AA patches who undergo corticosteroid injection experience hair growth, a rate that varies with disease severity, and the clinical response is generally perceived six weeks after starting treatment.^25^, ^27^, ^28^ Intralesional corticosteroid therapy can be applied to the scalp, eyebrows, beard, and other hairy areas of the body.^8^, ^22^ Although it is an important pillar of AA treatment, the ideal concentration and total dose of the drug to be applied are still being debated.^29^

Triamcinolone acetonide (TAc) is the most widely used injectable synthetic corticosteroid worldwide.^7^, ^30^ In Brazil, the available TAc is restricted in the package insert to intraocular use and, therefore, the most widespread form has become triamcinolone hexacetonide (TH), its less soluble derivative and with a greater risk of skin atrophy.^24^ Classically, a concentration of 2.5 to 10 mg/mL of triamcinolone is used for the scalp and 2.5 to 5 mg/mL for the face and other body areas.^31^, ^32^ A pilot study demonstrated that lower concentrations, such as 2.5 mg/mL, were as effective as higher concentrations and with a lower risk of atrophy.^33^ Another study showed that the best risk-benefit would be the use of 5 mg/mL on the scalp and lower doses for areas with a greater risk of atrophy.^34^ An alternative option to TH in the intralesional treatment of AA is betamethasone (betamethasone dipropionate 5 mg/mL + betamethasone disodium phosphate 2 mg/mL). To achieve dose equivalence with 2.5 mg/mL of triamcinolone, 0.05 mL of betamethasone should be diluted in 0.95 mL of 0.9% saline solution.^24^ A good response has also been reported with hydrocortisone acetate 25 mg/mL.^22^

Infiltration of 0.02 to 0.1 mL per point is performed in the dermis or upper portion of the subcutaneous tissue, with spacing of 0.5–1 cm between punctures and an interval of four to six weeks between sessions.^7^, ^13^, ^27^ The number of applications required for hair regrowth varies; a significant response is generally observed after three sessions. More sessions, at the physician discretion, may be necessary to achieve complete hair regrowth. Dilution with saline or glucose solution is recommended, which may or may not be mixed with lidocaine.^24^ Adding lidocaine may increase the risk of drug flocculation if the anesthetic contains methylparaben, propylparaben, or phenol as components of its vehicle, increasing the chance of atrophy.^24^ The amount of corticosteroid applied per session should not exceed 40 mg of triamcinolone or equivalent.^33^

Pain is a limiting factor, especially in children or patients with extensive forms of AA.^7^, ^28^ The use of topical anesthetics, vibration, or local cooling before application may be useful to minimize discomfort during the procedure.^35^ Needle-free devices are also an option, but the device must be sterilized before use.^7^ These complementary measures may allow infiltration in children and even in adults who initially reject intralesional therapy. Treatment should be discontinued if no improvement is observed after six months of starting the infiltrations.^7^, ^36^ Patients who do not respond to corticosteroids may show resistance due to low expression of thioredoxin-reductase 1 in the external root sheath of the hair follicle.^30^, ^36^

Adverse effects include pain and hemorrhage at puncture sites, headache, local skin atrophy, dyschromia, systemic absorption and, much more rarely, anaphylaxis.^13^, ^23^ Skin atrophy is common and usually reversible, but there is a risk of permanent damage. To minimize its risk, the suggestion is apply small volumes of the drug, more diluted concentrations, more spaced punctures and avoid superficial injections outside the correct plane of drug administration.^36^ Increased intraocular pressure and cataracts have been reported in patients undergoing multiple sessions of intralesional corticosteroid therapy for AA of the eyebrows.^22^

### Topical corticotherapy

Topical corticosteroid therapy is widely used in the treatment of all forms of AA, although its clinical efficacy is controversial due to limited evidence.^7^ It is usually used alone only in mild AA since its efficacy is apparently lower in more advanced forms of the disease.^25^ However, its use and possible benefits encompass all AA subtypes. The authors recommend topical corticosteroid therapy in patients who refuse intralesional therapy and in children, including in combination with systemic therapy in extensive cases.

Different local corticosteroids have been used in AA with variable responses.^36^ Comparative studies, however, have shown that very high-potency corticosteroids, such as clobetasol, are significantly more effective than lower-potency ones, such as hydrocortisone.^28^, ^37^ Among the advantages of this therapeutic modality are the fewer side effects compared to the systemic route and the possibility of its use in different vehicles.^31^ Clobetasol has already shown positive results when used in foam, cream or, ointment.^27^, ^31^ When applied under occlusion, its efficacy seems to be increased, with reports of some promising results even in severe forms of the disease, such as total and universal AA, although it may increase the chance of side effects.^27^, ^30^

The most common adverse effects are folliculitis, local skin atrophy, stretch marks, acneiform rash, telangiectasia, dyschromia and, rarely, adrenal suppression.^7^, ^36^ Washing the site 12 hours after application is recommended to reduce the incidence of folliculitis and a dosage of up to five times a week seems to prevent the appearance of atrophy.^7^

### Topical minoxidil

The action mechanism by which minoxidil stimulates the hair follicle has not yet been fully clarified. Vasodilation, angiogenesis, opening of potassium channels and stimulation of the proliferation of follicular dermal papilla cells are some of the proposed mechanisms.^38^, ^39^ By increasing the duration of the hair cycle anagen phase, minoxidil seems to have a reasonable application as soon as hair regrowth begins, with the aim of increasing the thickness and length of the newly regrown hair.

A meta-analysis found minoxidil to be 5% superior to placebo when used in patch AA. This evidence was classified as having moderate quality.^40^ The concomitant use of topical anthralin or intralesional corticosteroid therapy seems to provide superior results to single-therapy treatments.^41^ The expected side effects are hypertrichosis, contact dermatitis, and pruritus.^36^

Although controversial, topical minoxidil has been widely used in clinical practice as an adjuvant therapy in AA. It can be used mainly in localized forms that already present partial hair regrowth. The usual concentration for use in adults is 5%, in one or two daily applications.

### Topical immunotherapy

Topical immunotherapy (TIT) is performed with agents that trigger allergic contact dermatitis. They are used in AA treatment with the aim of reducing the lymphocytic inflammation in the anagen follicle. The mechanism is not well understood, but it is believed to be related to the diversion of the inflammatory process from the bulb to the site where the new inflammatory process is being induced by the medication. Thus, the follicle is able to recover.^42^, ^43^

Upon contact with patient skin, the sensitizer triggers a delayed hypersensitivity reaction (type IV), producing memory lymphocytes. Thus, after new exposure to the same agent, the immune system will produce a T-cell-mediated inflammatory response.

In Brazil, the most commonly used sensitizing agent for this purpose is diphencyprone, available only in compounding pharmacies. It must be formulated in acetone, which is highly volatile and must be kept in containers protected from light (amber-colored vials).

After the initial exposure to diphencyprone, the entire immunological cascade to elicit memory takes between two and three weeks. Only after this period will the substance be recognized as an allergen, causing dermatitis. The satisfactory clinical response can vary between 30%-48%, but if any response is considered, it can reach up to 72.2%.^42^, ^44^, ^45^ Substance use is divided into three phases: sensitization, follow-up, and maintenance. Each phase has its own peculiarities that will be discussed below.

#### Phase I (Sensitization)

In this phase, 2% diphencyprone diluted in acetone is used. A small amount is applied with a soaked cotton swab to a 2 × 2 cm area. The sensitization process can cause local hypochromia or hyperchromia, and therefore it is suggested that the application be made in a barely visible area, such as behind the ear, for example. It is recommended not to expose the area to sunlight or wash it for 48 hours.

#### Phase II (Follow-up)

After two to three weeks of phase I, phase II can be started. In this stage, diphencyprone is applied weekly starting with a low concentration and gradually increasing it.

This is the most delicate moment of the TIT, as it is necessary to establish the lowest concentration of diphencyprone that causes a cutaneous inflammatory process. The application should not be started with the same concentration as in the initial phase, as there is a high chance of causing exacerbated inflammation, leading to complications such as blisters.

The medication should be applied to half of the scalp in a sufficient quantity to slightly moisten the skin. One end of a soaked cotton swab is enough to spread the medication over half of the scalp.

It is therefore suggested to start with a low concentration and increase it progressively every two weeks: 0.01%; 0.02%; 0.05%; 0.1%; 0.2%; 0.5%; 1% and 2%. The progression speed is set at the physician discretion, and it can be done even more slowly or using intermediate concentrations in between those recommended above. The ideal concentration causes erythema, desquamation, pruritus and discomfort of mild to moderate intensity in the first 48 hours after the application. This means that it was sufficient to trigger the inflammatory process. Therefore, if the patient does not report any signs/symptoms after two applications, it is possible to increase the concentration in the third session. An alternative is that, in the same session, different concentrations can be applied to different quadrants of the scalp to more quickly identify the ideal dose for sequential applications of diphencyprone in the patient.

After reaching the ideal concentration, treatment should be maintained weekly. In cases of lack of response for six months, another treatment can be tried. However, some authors report the need to persist with therapy for up to 12-24 months to observe hair regrowth.^45^ If hair regrowth occurs, treatment should be maintained until acceptable cosmetic coverage without signs of activity are achieved.

#### Phase III (Weaning/maintenance)

After acceptable cosmetic hair regrowth, it is suggested to reduce the frequency of applications to biweekly, followed by monthly, and finally, discontinue treatment. In cases where the disease reactivates after stopping the medication, the possibility of continuing treatment fortnightly or monthly should be considered.

The most common complications are hyperpigmentation, hypopigmentation, severe eczema, blisters, lymph node enlargement, folliculitis, and flu-like symptoms.^44^

TIT is recommended as an alternative for severe AA without signs of activity or refractory localized AA, especially for those who cannot or do not wish to undergo systemic therapy. It is essential to ensure that the patient is capable of performing the treatment adequately and that the professional does not come into direct contact with the product during application due to the risk of sensitization.

### Anthralin

The use of anthralin also aims to divert the inflammatory process away from the bulb, but it causes irritant contact dermatitis, not allergic contact dermatitis. In Brazil, anthralin is only available through compounding, and like diphencyprone, it is often difficult to find. Its concentration can vary from 0.5‒2.0% diluted in cream. It causes hair regrowth in 71% of cases of patch alopecia^46^ and the average time for the initial response is three months, with full results after 15 months.^46^

The medication is applied to the affected area, penetrating up to 1 cm into the apparently healthy area. The patient is instructed to wash the area and remove the product thoroughly after 30 minutes. The procedure is repeated daily. The time of contact with the skin is increased by 15 minutes, every three days. In other words, it starts with 30 minutes, then increases to 45 minutes, 60 minutes, 75 minutes and so on, up to a maximum time of two hours. The aim is to cause mild eczema. If there is no reaction after two hours, the contact time can be extended, in some cases, to all night long.

Anthralin is a dark brown substance with a characteristic odor. With frequent applications, it ends up pigmenting the follicular ostia, simulating blackheads. However, dermoscopy clearly shows the difference between a black dot in the center of the ostium and the brown pigmentation regularly distributed around the edge of the follicular ostium caused by anthralin. One of the signs of therapeutic failure can be seen when no pigmentation of the ostia is observed, which can have three main causes: irregular application, short contact time with the scalp, or inadequate quality of the medication.

This is a safe therapy, but some precautions must be taken to avoid complications. Since the goal is to cause local irritation, the order of increasing the time the medication is in contact with the skin must be systematically followed, avoiding exacerbated irritation. The use of anthralin leads to hyperpigmentation of the treated area, but the color will return to normal after the treatment is interrupted. The use of corticosteroids is antagonistic to the effect of anthralin and diphencyprone because they reduce the inflammation anthralin and diphencyprone stimulate.

Anthralin is an option for children due to the absence of systemic side effects. It can be used as a second or third line of treatment for adults with mild AA, and even as an adjuvant in the treatment of severe AA.

### Systemic corticosteroids

Several forms of systemic administration of corticosteroids have been described for the treatment of AA. Since 1975, pulse corticosteroid therapy has been introduced in the treatment of AA, aiming at reducing side effects of long-term corticosteroid therapy and increasing therapeutic efficacy. Since then, several types of corticosteroid pulses have been described, especially for rapid rescue of severe and acute forms of AA.^36^

Oral mini-pulse dexamethasone was evaluated in a prospective cohort that used a mean dose of 2.7 mg/day, two days a week, in patients resistant to topical therapy. Approximately 51% of patients achieved SALT-50 after nine months of treatment. The daily dose used and treatment duration were associated with a higher frequency of side effects due to corticosteroid therapy. Hypothyroidism and early age of disease onset were predictors of lack of response to treatment.^47^

Oral pulse methylprednisolone was evaluated in two studies. In the first study, a mega dose of 15 mg/kg was evaluated in three different dosing regimens: three consecutive days every two weeks, two consecutive days every three weeks, and three consecutive days every three weeks, for 24 weeks. Regrowth rate was similar between the groups (>25%); however, the groups with shorter intervals had a higher frequency of side effects.^48^ The second study evaluated oral mini-pulse methylprednisolone 16 mg/day for two consecutive days per week. Forty percent of the participants showed satisfactory growth after three months, while more than 82% of the patients achieved the same results after six months.^49^

Mini-pulse prednisolone in severe AA was evaluated in a study that included 32 patients with SALT > 40 or persistent AA. Initially, the participants received a monthly pulse of 300 mg of prednisolone for four months. This dosage was effective for multifocal AA, with mild side effects. Refractory patients or those with universal AA responded with a pulse of 1000 mg/month. Females, disease duration greater than two years, and alopecia universalis were associated with poor or no response to treatment.^50^ Oral pulse prednisolone 200 mg/week for three months in patients with more than ten patches or with SALT ≥ 0 showed a response rate of approximately 34%.^51^ Other regimens have been described for patients with rapid progression or resistance, such as prednisolone 80 mg/day for three consecutive days every three months, and also the mega pulse of 15 mg/kg for two days every three weeks.^48^

There is no consensus in the literature on the dose and duration of daily oral corticosteroid therapy in AA. Prednisone can be used in doses ranging from 0.1 to 1 mg/kg/day. It is suggested to start with higher doses (0.5 to 1 mg/kg/day) and gradually reduce the dose (over six to 12 weeks) after hair regrowth is achieved.

Although it is not described in the guidelines for AA and has a higher cost, deflazacort has a more favorable safety profile. The drug has a high therapeutic index, with potency ranging from 70% to 90% of prednisone, and has a lower impact on calcium metabolism than any other synthetic corticosteroid. Moreover, it has a comparatively small effect on carbohydrate metabolism, water retention, and hypokalemia.^52^ It is suggested to start with a daily dose of 0.75 mg/kg for both adults and children and to reduce it slowly after hair regrowth.

Long-acting corticosteroids can be administered through the intramuscular or intravenous route as an alternative to oral use. The application of 40 mg of intramuscular TAc once a month for six months was shown to be superior to prednisolone pulse therapy (80 mg for three consecutive days every three months) and dexamethasone (0.5 mg/day for six months) in a randomized comparative clinical trial.^53^

As for intralesional use, TAc is also recommended over TH for intramuscular use due to the greater risk of developing atrophy and telangiectasias in the long term.^54^ The authors suggest, as a second option, replacement by the combination of betamethasone dipropionate 5 mg/mL (short-acting) with betamethasone disodium phosphate 2 mg/mL (long-acting).^24^ Table 4 shows the dose equivalence of the different corticosteroids.^55^

**Table 4:** Corticosteroid equivalence.

**Substance**	**Equivalent dose (mg)**	**Anti-inflammatory potency**	**Mineralocorticoid potency**	**Half-life (hours)**
**Cortisol (hydrocortisone)**	20	1	1	8‒12
**Cortisone**	25	0.8	0.8	8‒12
**Prednisone/ Prednisolone**	5	4	0.8	12‒36
**Deflazacort**	7.4	4	0.5	2
**Methylprednisolone**	4	5	0.5	12‒36
**Triamcinolone**	4	5	0	12‒36
**Betamethasone**	0.75	25	0	26‒72
**Dexamethasone**	0.75	25	0	36‒72
**Fludrocortisone**	‒	10	125	12‒36

Adapted from Caplan et al., 2007.^44^

Response rates with daily, intramuscular, or mini-pulse systemic corticosteroids are high, but many patients show recurrence when the dose is reduced or soon after the medication is discontinued. They are especially useful in controlling cases with rapid progression or in extensive cases with signs of activity. For those who respond to corticosteroids but become corticosteroid-dependent, the association of another systemic medication is necessary to spare its use.

Attention to the risk of side effects is essential for all types of long-term corticosteroid therapy. Endocrinological monitoring, bone densitometry assessment, and calcium and vitamin D supplementation are important measures to attenuate the risk of more severe adverse effects.^55^, ^56^, ^57^, ^58^

### Janus kinase inhibitors (JAKi)

JAKi were evaluated in double-blind, randomized, controlled, multicenter trials and were the first drugs officially approved for the treatment of severe AA. They inhibit the IFN-γ and IL-15 signaling pathways, which activate and perpetuate CD8+ cytotoxic T cells, responsible for autoimmune inflammation against the hair follicle.^59^

Baricitinib (JAK 1/2 inhibitor) and ritlecitinib (JAK 3/TEC inhibitor) are currently the two drugs approved in Brazil by Anvisa for the treatment of severe AA in patients aged ≥ 18 years and ≥ 12 years, respectively.

Baricitinib efficacy was demonstrated in two randomized phase 3 clinical trials involving 1,200 patients with severe AA (SALT > 50).^5^ At week 36, 38.8% of patients treated with baricitinib 4 mg/day achieved SALT < 20 in comparison with 22.8% of patients treated with 2 mg/day and 6.2% of patients receiving placebo. Extension studies with continuous baricitinib use for up to 104 weeks showed that efficacy continues to increase after 52 weeks and there is a high rate of maintenance of clinical results.^60^

In the phase 2b-3 study of ritlecitinib, patients with SALT > 50 were randomized to receive oral ritlecitinib or placebo with or without a four-week loading dose (200 mg loading dose followed by 50 mg or 200 mg loading dose followed by 30 mg, and 50 mg, 30 mg, 10 mg doses).^6^ At week 24, 31% of the patients in the ritlecitinib 200 mg/50 mg group, 23% of the patients in the ritlecitinib 50 mg group, and 2% of the patients in the placebo group achieved SALT ≤ 20. At week 48, 40% of the 200 mg/50 mg group and 43% of the 50 mg group achieved SALT ≤ 20, showing that the long-term outcome is similar between the two regimens.^61^

Other JAKi have shown efficacy in non-controlled studies and are considered options for off-label use, such as tofacitinib (JAK 1/3 inhibitor) at a dose of 5 mg twice daily, ruxolitinib (JAK 1/2 inhibitor) at a dose of 20 mg twice daily, and upadacitinib (JAK 1 inhibitor) at a dose of 30 mg/day. Topical preparations have not shown to be effective.^62^, ^63^ Deuruxolitinib was approved for the treatment of AA in 2024 by the FDA, and ivarmacitinib has a review application for approval.^64^, ^65^

To date, the use of JAKi in AA is considered safe. Adverse events that occurred more frequently than with a placebo were upper respiratory tract infections, urinary tract infections, nasopharyngitis, acne, increased transaminase levels, headache, and nausea. Adverse effects are generally mild and transient, and there was no increased risk of severe adverse effects when compared to placebo.^62^, ^63^

All JAK inhibitors have safety information in their package inserts from the tofacitinib safety study (ORAL Surveillance) carried out in patients with rheumatoid arthritis aged ≥ 50 years with one or more cardiovascular risks, which showed an increased risk of adverse cardiovascular events, venous thromboembolism, severe infections, malignant neoplasms, and death compared with TNF inhibitors, which have a cardioprotective effect.^66^ This risk was not observed in patients with immune-mediated dermatological diseases, including AA.^67^ However, until further studies are available, the treatment of elderly patients, smokers, or those at increased risk of these conditions should be evaluated with caution.^68^ Recommendations for the use of JAK inhibitors are summarized in Table 5 and pretreatment precautions in Table 6.

**Table 5:** JAK inhibitors in AA.

Baricitinib 4 mg/d and ritlecitinib 50 mg/d have a high level of evidence for severe AA and are approved by ANVISA for patients over 18 and 12 years old, respectively
Use is limited by the high cost
Other JAK inhibitors have been shown to be effective and may be off-label options
High recurrence rates after discontinuation require ongoing treatment
Side effects are generally mild, but it should be considered that there is little data on long-term safety.

AA, Alopecia Areata.

**Table 6:** Recommended care when indicating treatment with JAK inhibitors.

Not recommended for pregnant or lactating women
Assess the risk of thrombosis, neoplasia and cardiovascular events
Verify drug interactions
Pre-treatment tests: complete blood count, kidney function, transaminases and bilirubin (repeat after the first month and then every three months); lipid profile (repeat every three months)
Update vaccinations and consider vaccination for herpes zoster
Serology for hepatitis B, C and HIV (repeat after one year)
Quantiferon or PPD for tuberculosis (repeat after one year)
Chest X-ray (repeat after one year)
Treatment of latent tuberculosis, if indicated

### Methotrexate

Methotrexate (MTX) is a competitive inhibitor of dihydrofolate reductase. Its use at low doses in inflammatory diseases such as AA requires folic acid supplementation. Among the independent pathways, suppression of the JAK/STAT signaling pathway seems to be the main action mechanism.^69^

Initial doses of 5 to 10 mg/week are progressively increased, in four to six weeks, up to 20–25 mg. Since oral MTX at doses above 15 mg may show erratic absorption, the injectable option should be considered in these cases. The combination of oral and intralesional corticosteroids has been reported. In most cases, a minimum dose of MTX, ranging from 7.5 to 12.5 mg/week, is required for maintenance.^70^, ^71^

Better responses are observed in men, patients over 40 years of age, with less than five years of disease duration, who reached cumulative doses of 1000 to 1500 mg, and who received corticosteroids in addition to MTX.^71^, ^72^ Recurrence may occur during treatment and after discontinuation of the medication and long-term use is necessary.

MTX in combination with low doses of prednisone has shown terminal hair regrowth in up to 96% of patients with AA.^70^ Total regrowth has been demonstrated in 15% to 64% of the patients.^72^

Pancytopenia is the most feared toxicity when using low-dose MTX, but it is uncommon.^73^ Patients with renal failure, and hypoalbuminemia, who use erroneously high doses or use other medications that interact with MTX (e.g., anti-inflammatory drugs) are at greater risk.^74^, ^75^ Interstitial pneumonitis and altered liver function have been reported.^72^ Folic acid supplementation reduces the side effects of MTX, especially gastrointestinal ones. Different doses and frequencies of administration have been described, ranging from 5 mg per week to 1–5 mg/day. Even in daily doses, folic acid does not interfere with MTX efficacy.^76^ Folinic acid supplementation is reserved for drug toxicity cases.^76^

It is suggested that a chest X-ray, serology for hepatitis B, C, and HIV, complete blood count, beta-HCG, and renal and liver function tests be performed before starting the treatment. Complete blood count, renal and liver function tests, and beta-HCG should be repeated at day 30 and after 60 to 90 days according to clinical evaluation.

### Cyclosporine

Cyclosporine is an immunosuppressive agent capable of inhibiting the activation of helper T-cells and suppressing the production of interferon-gamma, reducing the perifollicular inflammatory infiltrate. The efficacy of cyclosporine was proven in a controlled, double-blind, and randomized study.^77^ A dose of 2 mg/kg/day divided into three doses is initially used, with a progressive increase up to 5 mg/kg/day.^78^ Hair regrowth varies from 25% to 76.6% when associated with systemic and intralesional corticosteroids.^78^, ^79^

Although effective, its use is limited by side effects, especially nephrotoxicity, immunosuppression, and arterial hypertension. Nephrotoxicity, generally due to prerenal vasoconstriction, although reported, was reversible in patients with AA.^78^, ^79^ The duration of cyclosporine use is limited and should be monitored. In severe cases, this can be challenging since when treatment is discontinued, AA tends to recur.

### Azathioprine

Azathioprine is an antimetabolite with few reports on its use in AA and low therapeutic response. Initial doses of 0.5‒1 mg/kg/day can be increased to 2‒3 mg/kg/day according to patient tolerance.^80^ At a dose of 2.5 mg/kg/day, it can be considered in recalcitrant AA. Some hair regrowth was observed in 43% of patients after four to six months.^80^ Gastrointestinal effects, elevated liver enzymes, pancreatitis, and bone marrow suppression are the most commonly reported side effects.

Better responses may be observed in combination with systemic or injectable corticosteroids or even MTX. The combination with MTX should be performed with caution, considering the increased side effects.^81^

### Oral minoxidil

The benefits of oral minoxidil in AA include anagen induction, aid in the treatment of coexisting androgenetic alopecia, and possible immunomodulation.^82^ The use of oral minoxidil in AA was first reported in the literature in 1987, in monotherapy with a dose of 5 mg, twice daily. Satisfactory hair regrowth occurred in 18% of the patients.^83^ Despite the high dose, facial hypertrichosis was observed in only 17% of the patients.

It was observed in a non-comparative study that oral minoxidil may have an additive effect when associated with JAK inhibitors.^84^ This association may benefit partial responders or even allow the use of lower doses of JAKi.

Oral minoxidil has been studied as monotherapy or adjunctive treatment in 363 patients with AA, including 42 children. Treatment duration ranged from four to 20 months. Daily doses ranged from 0.03 mg/day to 15 mg/day. Hypertrichosis was the most commonly reported adverse effect in all AA studies to date, ranging from 11.5% (0.99 mg) to 50% (2.5 and 5 mg for women and men, respectively). Mild cardiovascular adverse effects, including postural hypotension, palpitations, and peripheral edema, were less common. Severe cardiovascular adverse effects (pericardial effusion, tamponade, and angina pectoris) were not reported in any of these studies.^82^

Despite the limited evidence of results in monotherapy, oral minoxidil may be an interesting option as an adjunctive therapy, especially in extensive cases on systemic therapy.

### Excimer laser/light

The most studied light-based therapies in AA are excimer laser and 308-nm excimer light, which have immunosuppressive properties, possibly by inducing T-cell apoptosis. A review included eight clinical trials and case reports, with a total of 94 treated individuals, with an efficacy of 36.9% to 100%, with regrowth equal to or greater than 50%.^85^ A meta-analysis that included only four controlled studies using excimer laser on previously untreated AA patches confirmed the efficacy of the treatment.^86^ The main side effects of the therapy are mild erythema, pain during application, hyperpigmentation, blistering, pruritus, and desquamation.^87^ The high cost is the main disadvantage of the treatment. Excimer laser and excimer light may be treatment alternatives for mild and moderate refractory cases, especially when there is corticosteroid-induced atrophy or contact dermatitis due to other therapies.

### Prosthetics and camouflage

Cosmetic camouflage options should be encouraged. Although few studies have quantified the benefit in the patients quality of life, the use of these resources is considered good practice even during treatment periods.^88^, ^89^ There are numerous options for partial or total hair prostheses, and removable or fixed extensions and hairpieces.

Camouflage may be presented as hair fibers, sprays, waxes, and pigmented powders. Tricopigmentation may be performed on the scalp and eyebrow area. Eyelashes, false nails, and eyebrow prostheses may also be used.

## Treatment in special situations

### Alopecia areata in children

AA is a common cause of hair loss in childhood and adolescence. Up to 60% of cases begin in the first two decades of life.^90^ In children, especially in mild cases, topical corticosteroids, preferably medium to high-potency ones (mometasone 1% to clobetasol 0.05%), are usually employed as the first therapeutic option. When possible, occlusion should be performed at night (e.g., using a shower cap, swimming cap, or plastic wrap). When there is a contraindication or lack of response to topical corticosteroids, anthralin (0.5–1%), diphencyprone, or minoxidil may be used in combination.^46^, ^91^ Although effective, intralesional corticosteroid therapy is limited by the intolerance to pain and fear of needles that are common in this age group.

The administration of systemic medications may be considered in extensive cases or in cases of evident activity. The only medication with package insert approval for the systemic treatment of AA in the pediatric population is ritlecitinib, from 12 years of age.

The off-label use of JAK inhibitors in children under 12 years of age with severe AA has been described in case reports and small retrospective studies.^92^ To date, the literature suggests a good safety profile for the use of this category of medications in children. In a phase III clinical trial with 483 patients, baricitinib was shown to be safe and effective for the treatment of atopic dermatitis in children and adolescents between two and 18 years of age.^93^ Only 0.6% of the patients discontinued treatment due to adverse effects – none of them severe, compared to 1.6% of patients in the placebo group.^93^ Other studies evaluating the efficacy and safety of JAKi in childhood are ongoing, including a clinical trial with ritlecitinib for children aged six to 12 years with extensive/severe alopecia areata (Clinical Trial NCT05650333).

Among the other options available for systemic treatment, the authors prefer systemic corticosteroids and MTX (at a dose of 0.2 to 0.4 mg/kg/week). It is important to take into account the child stage of life and development and to be aware of possible adverse effects, especially changes in growth, metabolism, and immune competence.

### Alopecia areata in the beard area

The beard is the second most frequently affected area by AA, second only to the scalp.^8^ There are no randomized controlled clinical trials evaluating specific treatments for this region. The most common approach is the use of local therapies, starting with topical corticosteroids, followed by intralesional ones, or the direct use of intralesional corticosteroids, a therapy that has the greatest evidence of efficacy for localized and short-term AA.^94^ The dose of 2.5 mg/mL of triamcinolone is the most appropriate for treating the face. Although effective, the use of corticosteroids carries a greater risk of atrophy in this area. Minoxidil can be used as adjuvant therapy.

### Alopecia areata of the eyebrows and eyelashes

The involvement of the eyebrows and eyelashes has a major impact on quality of life, as it directly interferes with physiognomy. Alopecia in these specific areas is a factor that increases severity and may indicate systemic treatments. With baricitinib, approximately one-third of the patients with severe eyebrow and eyelash loss achieved normal or near-normal hair growth within 36 weeks of treatment, and with ritlecitinib, approximately 23% of patients with eyebrow or eyelash loss achieved normal or near-normal hair growth within 24 weeks.^95^ Topically, the only agents indicated for hypotrichosis or alopecia in the eyelash region, regardless of its etiology, are prostaglandin analogs, especially bimatoprost 0.03% solution.^96^, ^97^ Some studies have tested the drug in adult and pediatric patients, with variable results, which is probably due to different severity of the conditions and, therefore, different AA prognoses.^98^ It seems to be a safe and potentially effective agent for the treatment of the region. It is important to be aware of the risk of eyelid hyperpigmentation and darkening of the iris.

In addition to the possibility of using bimatoprost 0.03% for the eyebrows, it is also possible to use topical and intralesional corticosteroids.^96^ Preferably use medium-potency corticosteroids, taking care not to drip onto the eyelids and eyes (a cream vehicle should be preferred rather than a solution). Intralesional infiltration of triamcinolone should be performed at a dose of 2.5 mg/mL to minimize the risk of skin atrophy. The association with topical minoxidil is also an option, similar to the scalp.

### Alopecia areata and atopic dermatitis

Dupilumab was the first biological drug approved for the treatment of moderate to severe atopic dermatitis, and some studies have reported improvement in AA symptoms in patients who received it, raising the hypothesis that it is a useful drug for patients with both conditions. However, there are also reports of worsening AA symptoms or even the emergence of the disease in patients undergoing treatment, which leaves its benefit as an uncertain possibility.^99^

## Follow-up

The interval between visits will depend on the treatment regimen chosen for each patient. Those treated with diphencyprone will require weekly or biweekly visits; those undergoing intralesional injections should come every four to six weeks. Patients undergoing topical home treatment can come every two to three months, while those receiving systemic treatments will have intervals determined depending on the chosen drug and the patient health status. At the initial consultation and at least quarterly, patients should undergo standardized clinical photographs documenting different regions of the scalp (top, sides, occipital region). For JAK inhibitors, quarterly clinical and laboratory evaluations are recommended. Dermoscopic photographs of the alopecia areas are recommended. Classification using one of the methods described for estimating extent (e.g., SALT score) and assessment of ungual and body hair involvement should be included in the patient medical record. Other instruments, such as quality of life questionnaires, can be adapted for use in AA and are important when assessing therapeutic response.^100^

## Final considerations

The treatment of AA is complex because it depends on the extent and location of involvement, time of evolution, impact on quality of life, age, comorbidities, possible adverse effects of treatments and patient expectations. This consensus aims to provide basic guidelines for AA management in daily practice.

## Authors' contributions

Paulo Müller Ramos: Design and planning of the study, review of the literature, drafting and editing of the manuscript and final review of the manuscript.

Alessandra Anzai: Design and planning of the study, review of the literature, drafting and editing of the manuscript and final review of the manuscript.

Bruna Duque-Estrada: Design and planning of the study, review of the literature, drafting and editing of the manuscript and final review of the manuscript.

Daniel Fernandes Melo: Design and planning of the study, review of the literature, drafting and editing of the manuscript and final review of the manuscript.

Flavia Sternberg: Design and planning of the study, review of the literature, drafting and editing of the manuscript and final review of the manuscript.

Leopoldo Duailibe Nogueira Santos: Design and planning of the study, review of the literature, drafting and editing of the manuscript and final review of the manuscript.

Lorena Dourado Alves: Design and planning of the study, review of the literature, drafting and editing of the manuscript and final review of the manuscript.

Fabiane Mulinari-Brenner: Design and planning of the study, review of the literature, drafting and editing of the manuscript and final review of the manuscript.

## Financial support

None declared.

## Conflicts of interest

PMR (Aché, FQM, GSK, L’Oreal, Pfizer, Theraskin, USK: external guest on advisory board; guest speaker and participant at a sponsored event).

AA (Pfizer, USK: external guest on advisory board; Pierre-Fabre: speaker; Mantecorp: preparation of scientific material).

BDE (Pfizer: external guest on advisory board; guest speaker and participant at sponsored event).

DFM (Pfizer/Biolab/L’Oreal: external guest on advisory board; guest speaker and participant at a sponsored event).

FS (Biolab: guest speaker and participant at a sponsored event).

LDNS (Aché, FQM, GSK, Pfizer): external guest on advisory board; guest speaker and participant at sponsored event).

LDA (Pfizer, Aché: external guest on advisory board; Aché, Biolab, Valeant, L’Oreal, USK, FQM: speaker; USK: preparation of scientific material; USK and Genom: product evaluation in patients; Pfizer, GSK: guest participant at sponsored event).
